# Odoribacter splanchnicus—A Next-Generation Probiotic Candidate

**DOI:** 10.3390/microorganisms13040815

**Published:** 2025-04-03

**Authors:** Jianhong Li, Jing Xu, Xue Guo, Haoming Xu, Chen Huang, Yuqiang Nie, Youlian Zhou

**Affiliations:** 1 Department of Gastroenterology and Hepatology, The Second Affiliated Hospital, School of Medicine, South China University of Technology, Guangzhou 510006, China; jianhongli2023@163.com (J.L.); zyxujing1995@163.com (J.X.); guoxue0606@163.com (X.G.); haomingxu1992@126.com (H.X.); chenhuanghappy@outlook.com (C.H.); 2 Department of Gastroenterology and Hepatology, Guangzhou First People’s Hospital, South China University of Technology, Guangzhou 510180, China

**Keywords:** *Odoribacter splanchnicus*, human health, next-generation probiotics, nutrition

## Abstract

As an important intestinal microorganism, *Odoribacter splanchnicus* frequently appears in high-throughput sequencing analyses, although pure culture research on this microorganism is not as advanced. It is widely present in the mammalian gut and is closely associated with the health status of the host and the incidence of various diseases. In recent years, changes in the abundance of *O. splanchnicus* have been found to be positively or negatively correlated with health issues, such as obesity, metabolic syndrome, diabetes, and intestinal inflammation. It may exhibit a dual protective or promotional role in specific diseases. Thus, it may play an important role in regulating host metabolism, immune response, and intestinal homeostasis. Additional research has revealed that *O. splanchnicus* can synthesize various metabolites, especially short-chain fatty acids (SCFAs), which play a key role in promoting intestinal health, enhancing energy metabolism, improving insulin resistance, and regulating immune responses in the host. Therefore, *O. splanchnicus* is a strong candidate for “next-generation probiotics”, and its potential probiotic function provides novel ideas for the development of functional foods and the prevention and treatment of metabolic and intestinal inflammatory diseases. These findings can help develop new biological treatment strategies and optimize health management plans.

## 1. Introduction

In the past two decades, the revolutionary progress in high-throughput sequencing technology has significantly improved our understanding of the gut microbiota. We have achieved unprecedented accuracy in species identification and gradually uncovered numerous unknown “microbial dark zones” in the gut [1]. With advancements in sequencing methods, from 16S rRNA amplicon sequencing technology to advanced high-throughput sequencing technology used in metagenomics, our understanding of the complexity and diversity of intestinal microorganisms has progressively deepened. The gut microbiota has emerged as a pivotal environmental factor in regulating host health, with alterations in its composition and activity being closely linked to the onset of various diseases [2]. Next-generation probiotics (NGPs) refer to a new class of probiotic strains that offer enhanced benefits compared to traditional probiotics [3,4]. These benefits may include improved survival rates in the gastrointestinal tract, enhanced adhesion to intestinal epithelia, superior immunomodulatory effects, or the ability to produce beneficial metabolites not commonly associated with conventional probiotics [5,6]. NGPs are designed to address specific health needs or disease states with greater precision and efficacy. In this context, *Odoribacter splanchnicus*, a newly emerging gut bacterium of interest, has been frequently identified in various studies on the gut microbiome. Its pure culture system is yet to be established, and its metabolic mechanism and biological characteristics need to be elucidated. Existing research data suggest that a high abundance of *O. splanchnicus* is significantly positively correlated with healthy intestinal environmental characteristics, such as a preference for low-fat diets and a lean body status [7]. This indicates that this bacterium may play an important role in maintaining human health. Concurrently, multiple studies have also revealed the complex and close relationship between the presence of *O. splanchnicus* and a series of disease states, especially inflammatory diseases [8,9]. This highlights the research value of the bacterium as a potential health regulator. High-throughput sequencing analysis of the gut and fecal microbiome revealed that *O. splanchnicus* accounts for a large proportion of the human gut microbial community. This finding has not only reinforced the central role of the bacterium in maintaining the ecological balance of the gut but also increased interest in its potential health benefits. Of note, *O. splanchnicus* was shown to produce various short-chain fatty acids (SCFAs), with butyrate as a representative molecule. These molecules exert wide and profound effects on intestinal health, immune regulation, and energy metabolism. An in-depth analysis of the complete genome sequence of this bacterium revealed that the expression of only approximately 61% of its protein-coding genes can be clearly associated with predicted biological functions [10]. This indicates the presence of some unknown or unexplored areas in the functional annotation of this strain’s genome. Additional research data are warranted to determine its precise characteristics.

Given the aforementioned findings, exploring the potential of *O. splanchnicus* as an important new-generation probiotic candidate, similar to *Faecalibacterium prausnitzii*, *Akkermansia muciniphila*, and *Halomonas* spp., represents a research direction with both challenges and opportunities. In the future, an in-depth analysis of its metabolic pathways, ecological functions, and molecular mechanisms of interaction with the host can help us develop novel probiotics and promote human health. In this review, we comprehensively summarize the information available on *O. splanchnicus*, including its historical background, the current understanding of its association with health and disease, underlying mechanisms of action, and its prospects in future research and applications.

## 2. Isolation and Major Properties

**Figure 1:**
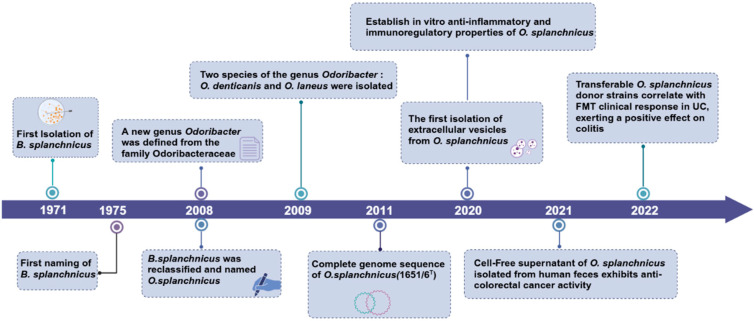
Timeline of major advances related to *Odoribacter splanchnicus*. Created in BioRender website by Li, J. (2025) and are available at the: https://BioRender.com/4zgdlc7 (accessed on 27 March 2025).

*O. splanchnicus* is a bacterium of significant research value. It was initially isolated in 1971 and named in 1975 by Werner et al. [11], who isolated it from abdominal abscess samples. Owing to its numerous shared characteristics with members of the *Bacteroides* genus, it was initially named *Bacteroides splanchnicus* [12]. With continuous advancements in bacterial taxonomy, in 2008, a new genus, *Odoribacter*, was defined within the Odoribacteraceae family of the Bacteroidales order. Compared to *Bacteroides fragilis*, *B. splanchnicus* exhibited numerous unique biochemical features, and the homology correlation between their 16S rRNA genes was less than 20% [13]. Consequently, *B. splanchnicus* was reclassified as a novel species within the *Odoribacter* genus [14,15] and was officially named *O. splanchnicus*. This genus comprises two additional species: *Odoribacter denticanis*, isolated from canine periodontitis [14], and *Odoribacter laneus*, isolated from human feces [16]. *O. splanchnicus* was also isolated from the blood and peritoneal pus samples of a patient with pelvic peritonitis [17] (Figure 1).

At the species level, *O. splanchnicus* is a strictly anaerobic, gram-negative bacillus with a short rod shape. It occurs as individual cells or loosely aggregated groups, and it is non-pigmented and non-sporulating [10]. It thrives best under anaerobic conditions at 37 °C, relying on chemical organic substances as its primary source of nutrition. This bacterium possesses a broad metabolic capability, participating in the synthesis and metabolism of secondary bile acids. It can ferment various sugars, including glucose, fructose, galactose, arabinose, lactose, and mannose, which are abundant in the gut and serve as substantial sources of carbon for the growth of the strain. However, *O. splanchnicus* cannot use specific sugars, such as sucrose, rhamnose, trehalose, or salicin [12]. When amino acids are the sole carbon source available, this strain specifically uses lysine to produce butyrate [12], while minimally affecting the production of other SCFAs, such as acetate, propionate, isovalerate, and valerate [18]. Another study revealed that the mono-colonization of *O. splanchnicus* in mouse cecal contents significantly increased the levels of acetate, butyrate, and propionate, which helped generate SCFAs through GPR43 and GPR109a receptors [8]. Additionally, as a member of the Odoribacteraceae family, *O. splanchnicus* may produce secondary bile acids with antimicrobial properties, such as isoallolithocholic acid [19]. This strain synthesizes various enzymes, including phosphatases, β-galactosidases, α-fucosidases, N-acetyl-β-D-glucosaminidases, and glutamate decarboxylases, which play crucial roles in the metabolism of carbohydrates and amino acids. Notably, *O. splanchnicus* also possesses highly active enzymes related to the pentose phosphate pathway, such as glucose-6-phosphate dehydrogenase and 6-phosphogluconate dehydrogenase. These help it maintain intracellular redox balance and energy metabolism [20].

*O. splanchnicus* exhibits varying degrees of sensitivity to multiple antibiotics [21]. It is highly susceptible to tetracyclines, lincomycin, clindamycin, rifampicin, and erythromycin but strongly resistant to aminoglycosides and polymyxins. These varying susceptibilities may be attributed to the unique structure of its cell wall or specific resistance mechanisms. Furthermore, combination therapy with ciprofloxacin and metronidazole antibiotics was shown to inhibit its growth [21].

## 3. Factors Influencing the Abundance of *O. splanchnicus*

Although *O. splanchnicus* does not rely on heme for its growth, the presence of heme significantly promotes its growth [12]. Therefore, heme may serve as an important growth factor or signaling molecule for this strain. Beyond its unique non-heme-dependency but heme-stimulated growth, we must acknowledge the subtle connection between its abundance and the dietary habits of the host. Notably, heme, a key component in red meat, may indirectly facilitate the proliferation of *O. splanchnicus* in the gut. A positive correlation has been reported between *O. splanchnicus* abundance and red meat consumption, whereas a negative correlation has been reported with vegetable intake [22]. Notably, in dietary contexts, the adoption of a low-calorie, low-carbohydrate, high-fat, weight-loss diet is associated with a significant increase in the abundance of this bile-tolerant bacterium [23]. The enrichment of *O. splanchnicus* varies among populations and is influenced by genetics, environment, diet, and other factors. For instance, it is enriched in the gut of Dutch individuals, and its abundance differs significantly from that in South Asian Surinamese populations, who exhibit a higher prevalence of type 2 diabetes mellitus and the absence of certain bacteria capable of producing SCFAs, which may include *O. splanchnicus* [24]. Additionally, bile, an essential component of intestinal digestive fluids, promotes the growth of *O. splanchnicus*, and the addition of 20% bile further optimizes its growth environment [25]. In investigations on animal models of specific disease-related conditions, probiotics and prebiotics have effectively promoted the growth and balance of beneficial gut microbiota and exerted a positive impact on gut health [26,27,28]. Certain probiotics or prebiotics can significantly increase the abundance of *O. splanchnicus* by modulating the gut microecological balance. For instance, the treatment of hyperuricemic mice with *Bacillus subtilis* fermented *Astragalus membranaceus*, a prebiotic or probiotic complex, not only alleviated disease symptoms but also significantly increased the number of *O. splanchnicus* in the gut [29]. Chen et al. [30] used metagenomic analysis to demonstrate that supplementing Apc^Min/+^ mice with *Lactobacillus plantarum* CCFM8661 significantly increased the abundance of *O. splanchnicus* in the gut and alleviated colorectal cancer (CRC) symptoms by restoring the intestinal barrier [30]. Etxeberria et al. [31] reported that adding Pterostilbene to the standard diet of genetically obese murine models exerted protective anti-obesity effects, improved insulin sensitivity, induced structural changes in the gut microbiota composition, and significantly increased the abundance of *O. splanchnicus*, which was also negatively correlated with fat mass in the mice. A study on the preventive effects of lactic acid bacteria-based probiotics on intra-abdominal hypertension-mediated intestinal barrier injury showed that pretreatment with a single-strain probiotic (L92) significantly increased the abundance of *O. splanchnicus* [32]. However, not all probiotics can directly or indirectly increase the abundance of *O. splanchnicus*. This suggests that the upregulation or downregulation of its abundance may be strain- or prebiotic-specific.

## 4. The Multifaceted Roles of *O. splanchnicus* in Host Health

**Figure 2:**
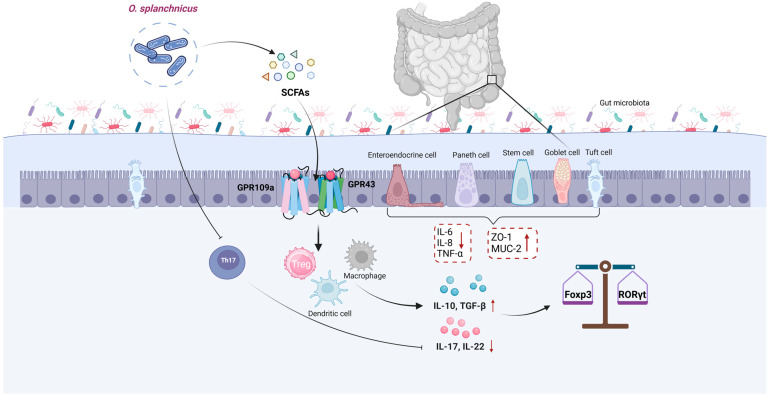
The potential mechanisms through which *Odoribacter splanchnicus* is involved in gut health. Created in BioRender website by Li, J. (2025) and are available at https://BioRender.com/6e3zb29 (accessed on 27 March 2025).

In studies exploring the multidimensional effects of gut microbiota on host health, *O. splanchnicus*, a key intestinal bacterium, has attracted attention due to its ecological and functional properties (Figure 2). Notably, its high abundance in Tak1^ΔM/ΔM^ mice and its ability to induce the development of intestinal Th17 cells [18] indicate its functional relevance even in the absence of immune system components. Th17 cells, as a vital immune cell type, play a pivotal role in combating certain infections and modulating autoimmune responses. By inducing Th17 cells, *O. splanchnicus* could impart resistance to chemically induced colitis and CRC in both conventional and germ-free mice [18]. Further research highlights that, beyond Th17 cell induction, *O. splanchnicus* primarily stimulates IL-10 production in immune cells. In vitro studies show that this bacterium releases outer membrane vesicles or produces specific metabolites, which trigger immune cells to generate the anti-inflammatory cytokine IL-10, thereby exerting an anti-inflammatory effect in the gut [9]. Lima et al. [8] demonstrated that *O. splanchnicus* metabolizes butyrate to promote the proliferation of Foxp3/RORγt regulatory T cells and induce IL-10 production through lymphocyte-dependent pathways, thereby reducing inflammatory responses. Additionally, *O. splanchnicus* strengthens intestinal mechanical and mucosal barriers by upregulating ZO-1 and MUC2 expression, promoting goblet cell production [33], and modulating IL-10 levels to enhance intestinal immunity and alleviate CRC symptoms. Another study revealed that malate, a metabolite found in *O. splanchnicus* culture supernatants, can induce apoptosis in early-stage colon cancer cells [34]. However, while *O. splanchnicus* is predominantly reported to benefit host health, its role in specific contexts, such as the gut microbiota composition in CRC patients, appears more complex. Han et al. [35] found that a high abundance of this bacterium in patients with non-immune-type CRC correlated with adverse clinical outcomes, including increased vascular and lymphatic infiltration and shorter overall survival. Other studies have reported lower bacterial richness and diversity in CRC patients compared to healthy controls, along with elevated levels of pro-carcinogenic bacteria like *Bacteroides fragilis* and *O. splanchnicus* [36]. Cai et al. [37] also identified *O. splanchnicus* as a novel driver species in CRC.

The dual-faced nature of *O. splanchnicus* can be attributed to several factors. In a healthy gut environment, the immune system is well-regulated, and *O. splanchnicus* can interact with the host’s immune cells in a balanced manner. For example, it induces the development of Th17 cells, which help defend against pathogens and maintain immune homeostasis [38]. The production of IL-10 stimulated by *O. splanchnicus* also contributes to the anti-inflammatory state of the gut, preventing excessive immune responses [39]. However, in the context of CRC, the situation differs. The tumor microenvironment is often immunosuppressive [40,41], and the normal immune-modulating functions of *O. splanchnicus* may be disrupted. The high abundance of *O. splanchnicus* in non-immune-type CRC patients may be associated with an altered immune response. It might interact with other components of the gut microbiota and tumor cells in a way that promotes tumor progression. Moreover, genetic and epigenetic variations [42] in the host’s cells can influence the host’s response to *O. splanchnicus*. Genetic differences in immune-related genes [43] may affect the host’s immune system’s ability to recognize and respond to *O. splanchnicus* appropriately, leading to different disease progression outcomes. In addition, the overall composition of the gut microbiota plays a crucial role. *O. splanchnicus* does not act in isolation. In a healthy gut microbiota, it may interact with other beneficial bacteria to maintain gut homeostasis [44]. However, in the dysbiotic gut microbiota often observed in CRC patients, the balance of these interactions is disrupted [45]. The presence of other pro-carcinogenic bacteria may synergize with *O. splanchnicus* to create a more favorable environment for tumor growth. Therefore, a deeper understanding of the mechanistic roles of *O. splanchnicus* in different health states is essential for developing novel therapeutic strategies for intestinal diseases.

## 5. A Study on the Association Between *O. splanchnicus* and Various Disease States

**Figure 3:**
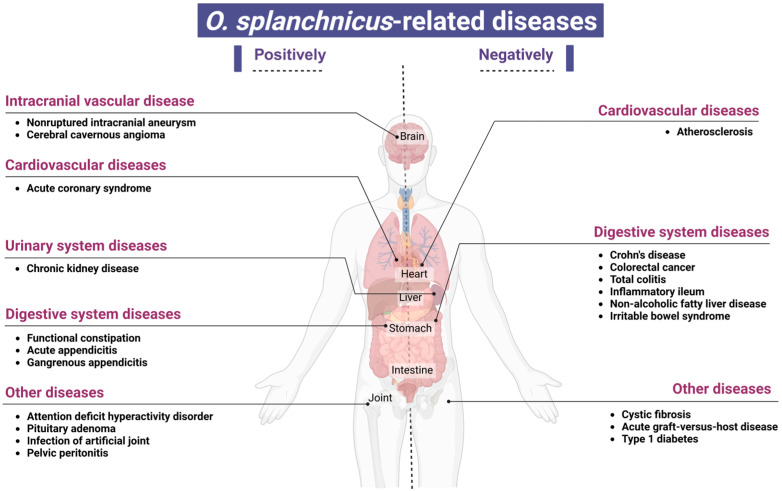
The variation in the abundance of *Odoribacter splanchnicus* correlates positively or negatively with certain diseases. Created in BioRender website by Li, J. (2025) and are available at https://BioRender.com/w2xh6xj (accessed on 27 March 2025).

The use of high-throughput sequencing technology has led to the frequent identification of *O. splanchnicus* in detailed investigations of gut microbiota composition and its relationship with diseases. Notably, the population density of *O. splanchnicus* increases or decreases distinctly, manifesting as significant positive or negative fluctuations in various diseases. Here, we summarize the disease types that are closely associated, positively or negatively, with the presence of *O. splanchnicus*, with an aim to provide deeper insights into the role of gut microbiota in disease mechanisms (Figure 3).

## 6. Diseases Negatively Associated with the Presence of *O. splanchnicus*

Recent research has consistently depicted a close correlation between the reduced relative abundance of *O. splanchnicus* in the gut and various pathological states, offering a novel perspective on the intricate interplay between microbiota and host health. Specifically, in a study on compositional and functional changes in the gut microbiota in women with type 1 diabetes (T1D) during pregnancy, the authors observed notable microbial community remodeling, which tended to exacerbate inflammatory responses. These manifested as significant elevations in fecal calprotectin and intestinal fatty acid-binding protein levels and a marked decrease in the abundance of *O. splanchnicus* (FDR = 0.098), among other bacterial genera [46]. These alterations may elevate the risk of pregnancy-related complications in women with T1D. A multicenter prospective cohort study with the long-term follow-up of over 1500 hospitalized patients revealed the potential benefits of *O. splanchnicus* presence and its relation to *Ruminococcus* spp. (e.g., *Ruminococcus bromii*) in resistance to *Clostridium difficile* infections in hospital settings [47]. The presence of *O. splanchnicus* during the post-transplant engraftment period has been associated with a reduced risk of acute graft-versus-host disease (aGVHD), suggesting its potential role in preventing or mitigating aGVHD [48]. The decreased abundance of *O. splanchnicus* is closely linked to the pathological states of specific gut diseases, including Crohn’s disease [49,50], inflammatory ileum, CRC, and pancolitis [51]. In a study on microbiological changes in pediatric patients with Crohn’s disease undergoing infliximab treatment, a significant and sustained increase in the abundance of *Odoribacter* and other SCFA-producing bacteria was observed compared to that in controls [50]. Notably, *O. splanchnicus*, which can produce anti-inflammatory SCFAs (e.g., acetate, propionate, and butyrate) [10], exerts anti-inflammatory effects on the host. Thus, the reduction in *O. splanchnicus* abundance may influence host inflammatory responses because it reduces the levels of SCFAs. This contributes to the exacerbation and chronicity of Crohn’s disease and pancolitis. Further evidence suggests a negative correlation between *O. splanchnicus* presence and carotid plaque formation. Wang et al. [52] found that this species may indirectly slow down plaque development by reducing the circulating levels of inflammatory markers, such as chemokine (C-X3-C motif) ligand 1. The abundance of *O. splanchnicus* may be significantly low or the bacterium may be completely eliminated in gut microbiota samples from patients with atherosclerosis, with the abundance of the bacterium negatively correlated with the status of the disease [53]. A decrease in *O. splanchnicus* abundance is also observed in several other disease states. For instance, a decline in microbial diversity in patients with cystic fibrosis (CF) is accompanied by a significant reduction in the abundance of this species, further emphasizing its pivotal role in maintaining a healthy gut microbiota balance [54]. Similarly, the reduced abundance of *O. splanchnicus* is noted in patients with non-alcoholic fatty liver disease (NAFLD) [55] and irritable bowel syndrome (IBS) [56]. Notably, in patients with IBS, the abundance of *O. splanchnicus* is positively correlated with the levels of dihydrofolate, a crucial intermediate in folate synthesis. The prevalent low folate levels in patients with IBS suggest a potential role for *O. splanchnicus* in maintaining intestinal metabolic balance and nutrient absorption. Moreover, a significant decrease in *O. splanchnicus* abundance is observed in children with high body weight who have NAFLD [57]. Chen et al. [58] reported that *O. splanchnicus* and indolelactic acid are enriched in patients with ulcerative colitis who achieve clinical remission through encapsulated fecal microbiota transplantation (FMT). This enrichment is associated with the clinical remission of UC. Conversely, Ma et al. [59] conducted the metagenomic sequencing of pangolin feces and found that *O. splanchnicus* is more abundant in diseased pangolins than in healthy ones. These findings indicate that the abundance of *O. splanchnicus*, as a multifunctional gut bacterium, is closely associated with various disease states. It not only influences intestinal inflammation and metabolic activities but also potentially modulates systemic inflammatory and metabolic processes, thereby exerting profound effects on overall host health.

## 7. Diseases Positively Associated with *O. splanchnicus* Presence

Certain bacteria, through their metabolic activities and interactions with the host, exert positive effects on host health and are classified as probiotics when present in the gut. However, in different diseases, owing to changes in environmental conditions, competition with other microorganisms, and variations in host immune responses, these bacteria may trigger infections, inflammation, and other adverse reactions, thereby adversely impacting host health [60,61,62]. Chen et al. [63] used whole-genome shotgun sequencing to analyze the fecal microbiota of 117 untreated patients with systemic lupus erythematosus (SLE), of which 52 also provided pre- and post-treatment samples, and compared these with 115 gender- and age-matched healthy controls. They found that *O. splanchnicus* was significantly enriched in the guts of untreated patients with SLE. The peptides produced by this bacterium resemble the epitopes of the characteristic autoantibody Sm antigen in patients with SLE. This leads to misrecognition by the immune system and could potentially trigger or exacerbate immune abnormalities in patients with SLE. Li et al. [64] conducted a metagenomic sequencing analysis and comparison of the gut microbiota in patients with unruptured intracranial aneurysms and controls in a case–control study. The heterogeneity in the gut microbiota structure was significantly reduced in patients with intracranial aneurysms, with *O. splanchnicus* being significantly enriched in the aneurysm group. This indicated a potential link between the presence of this bacterium and the pathogenesis of intracranial aneurysms. The abundance of *O. splanchnicus* in the guts of patients with cavernous angiomas was significantly higher than that in healthy controls. Thus, a greater abundance of this bacterium in the gut may serve as a biological marker for CA, and dynamic changes in its abundance may be closely related to CA onset and progression [65]. Research on attention-deficit hyperactivity disorder (ADHD) has also revealed significant abnormalities in the gut microbiota composition of affected individuals, particularly the significantly greater abundance of *O. splanchnicus*, *Bacteroides caccae*, *Paraprevotella xylaniphila*, and *Veillonella parvula* in children with ADHD than in age-matched healthy children [66]. Gkougka et al. [67] and Wan et al. [24] showed that the abundance of *O. splanchnicus* is significantly higher in patients with ADHD and potentially affects neurotransmitter synthesis, dopamine metabolism, and the regulation of inflammation and neurodevelopment through cytokine release. The abundance of *O. splanchnicus* in the gut microbiota of patients with acute coronary syndrome (ACS) was significantly greater than that in the gut microbiota of clinically matched controls. This indicated a significant correlation between the presence of this bacterium and ACS. The bacterium may serve as a novel target for ACS risk assessment or intervention [68]. Gryp et al. [69] isolated and quantified the gut bacteria responsible for the production of uremic toxin precursors in patients with chronic kidney disease. They found that *O. splanchnicus*, through its specific metabolic mechanisms, produces phenylacetylglutamine, indole, and p-cresol, which are important markers of impaired renal function. This bacterium may play a significant role in the decline of renal function or accumulation of uremic toxins. Furthermore, *O. splanchnicus* presence exhibited a positive correlation with functional constipation and comorbid depressive and anxiety symptoms, particularly in relation to serum 5-hydroxytryptamine levels [70]. Lin et al. [71] observed a significant enrichment of *O. splanchnicus* in the gut of patients with growth hormone-producing pituitary adenoma. In animal model studies, glut1 conditional knockout mice exhibited fat accumulation, impaired glucose tolerance, and gut microbiota disturbance, including an increase in the abundance of *O. splanchnicus* and *Odoribacter laneus*, owing to the absence of glut1 activity in intestinal epithelial cells. This finding provides important insights into the interplay between metabolic diseases and gut microbiota [72].

Under normal circumstances, *O. splanchnicus* is a normal gut flora. However, under specific conditions, such as intra-abdominal inflammation and impairment of the intestinal barrier, it may act as an opportunistic pathogen and cause an infection. In a recent case report using blood culture methods, *O. splanchnicus* was confirmed as the causative pathogen in bacteremia, secondary to acute appendicitis. This indicates the potential pathogenicity of the bacterium [73]. Lara-Taranchenko et al. [21] identified *O. splanchnicus* as the causative pathogen in a case of prosthetic joint infection by isolating anaerobic gram-negative bacilli and confirming its identification using 16S rRNA gene sequencing. Several other clinical cases of human *O. splanchnicus* infections have been reported [17,74], including a case of bacteremia, a case of gangrenous appendicitis with bacteremia in an immunocompetent adult, and a case of pelvic peritonitis. Bennion et al. [75] detected *O. splanchnicus* in the peritoneal fluid, appendiceal tissue, and abscess contents in 12 of 30 cases of perforated or gangrenous appendicitis, along with other aerobic and anaerobic bacteria. However, despite several case studies indicating that *O. splanchnicus* can cause infections outside the gut system, including bacteremia, its precise role in the pathological process of intra-abdominal infections requires investigation and validation, with the specific mechanism of action yet to be clearly defined.

In summary, those studies reveal a significant association between the abundance changes in *O. splanchnicus* across various disease states and the onset or progression of diseases. However, it is crucial to emphasize that this association arises from the interplay of multiple factors, including diet, lifestyle, genetic background, and other environmental influences. Consequently, we cannot attribute the presence of *O. splanchnicus* alone as the direct cause of disease occurrence. Instead, these findings underscore the intricate relationship between gut microbiota and host health, while also providing direction for future research to further explore the mechanisms of interaction among these factors. Such investigations are essential for a more comprehensive understanding of disease pathogenesis, early diagnosis, and the development of effective intervention strategies.

## 8. Biomarker

*O. splanchnicus*, as a member of the microbial community, exhibits significant differences in its abundance, distribution, and functional activity under various disease states, suggesting its potential as a biomarker with crucial roles in disease diagnosis, prognosis assessment, and treatment efficacy monitoring. Li et al. [76] identified *O. splanchnicus* as one of the two key species associated with the occurrence or progression of hepatocellular carcinoma (HCC). It demonstrated superior performance in HCC diagnosis compared to alpha-fetoprotein. Additionally, in a large-scale study on a Chinese sample population (2262 individuals), differences in gut microbiota compositions between obese and normal-weight individuals were analyzed. *O. splanchnicus* was highlighted as one of the three potential biomarkers associated with obesity [77]. Lv et al. [78] found that certain characteristics or the presence of *O. splanchnicus* were associated with an increased risk of kidney cancer (KC), suggesting its role as a risk factor and a potential biomarker for KC assessment and diagnosis. Zhou et al. [79] used functional analysis and species-level comparisons to show that the abundance of *O. splanchnicus* in patients with pancreatic ductal adenocarcinoma (PDAC) was significantly different from that in healthy controls and patients with autoimmune pancreatitis (AIP), making it a potential biomarker for distinguishing patients with PDAC from healthy individuals and patients with AIP. Li et al. [80] used ecological and network analyses to show that the presence or abundance of *O. splanchnicus* in patients with UC may be related to their disease status. They identified *O. splanchnicus* as one of four “opportunistic pathogens” with potential diagnostic and therapeutic significance in UC, positioning it as a biomarker or therapeutic target for UC. In conclusion, altered *O. splanchnicus* abundance is not only a hallmark of gut microbial imbalance under various disease states but also a vital biomarker that may be helpful for regulating the health status of the host and preventing specific diseases.

## 9. Potential Applications of *O. splanchnicus*

As a crucial gut microorganism, *O. splanchnicus* has garnered increasing attention for its role in various health states and the progression of various diseases. A 16S rRNA and metagenomic sequencing study involving 32 longevity families revealed that the gut microbiota of centenarians was more diverse than those of younger and generally elderly individuals. The microbiota was characterized by a significantly greater abundance of anti-inflammatory bacteria and potential probiotics, particularly with a notably greater abundance of *O. splanchnicus* [81]. Sun et al. [82] used Spearman correlation analysis and FDR correction (q < 0.05) in a Japanese centenarian cohort study to confirm that the abundance of *O. splanchnicus* increases with age and is closely associated with the natural aging process, further supporting the potential influence of gut microbial composition on lifespan. Individuals with obesity, which is a major health challenge in modern society, exhibit distinct gut microbial characteristics. Compared to individuals at a normal body weight, individuals with obesity have significantly poor gut microbial diversity. They also have a markedly lower abundance of *Odoribacter splanchnicus*, which is a crucial producer of SCFAs [7]. The abundance of certain probiotics, such as *Akkermansia muciniphila*, which can prevent metabolic disorders like obesity and diabetes, also declines notably in the guts of individuals with obesity. Given the pivotal roles of SCFAs in regulating energy metabolism, enhancing gut barrier function, and exerting anti-inflammatory effects, restoring or increasing the abundance of *O. splanchnicus* may be a novel strategy for improving obesity. Moreover, the potential of *O. splanchnicus* in cancer treatment and prevention cannot be overlooked. Its role in modulating the gut microbial balance and promoting a healthy microbial ecosystem could help reduce cancer risk and enhance therapeutic outcomes. Its potential highlights its significance in the broader context of gut health and disease prevention. Pan et al. [83] discovered that specific drug treatments or FMT could help increase the abundance of microbiota such as *O. splanchnicus* and enhance the host’s anti-tumor immune functions, including the activation of NK and T cells. This finding presents a novel approach to cancer therapy via gut microbiota modulation to bolster the anti-tumor capability in the host. Patients with acute myeloid leukemia undergoing intensive treatment exhibit intestinal barrier dysfunction and reduced microbial diversity, which includes a decrease in the abundance of specific taxa like *O. splanchnicus*. This emphasizes the importance of maintaining a gut microbial balance in cancer therapy [84]. Moreover, alterations in the gut microbiome of patients with chronic pain offer a new perspective on the application of *O. splanchnicus*. Significant changes are noted in the gut microbiome of patients with chronic pain, characterized by lower alpha-diversity and relative abundance of certain bacterial populations, including *O. splanchnicus* [85]. These changes may influence pain perception and modulation through complex mechanisms involving the gut–brain axis. These findings suggest that gut microbiota modulation, particularly increasing the abundance of *O. splanchnicus*, could help identify novel therapeutic targets for chronic pain relief. *O. splanchnicus* exhibits superior anti-inflammatory properties and immunomodulatory effects. To better apply the properties of the bacterium, Bosch et al. [86] successfully encapsulated *O. splanchnicus* strains into tablets using freeze-drying technology, direct compression, and fluidized bed coating, ensuring their viability throughout the manufacturing process. They confirmed that the strain could reduce IL-8 cytokine release from HT-29 cells induced in response to *Escherichia coli* lipopolysaccharide, a key inflammation indicator. Additionally, extracellular vesicles containing bioactive molecules with anti-inflammatory effects have been successfully isolated and extracted from *O. splanchnicus*, and their immunomodulatory role in vitro has been demonstrated [9,87]. In the future, microecological preparations focusing on *O. splanchnicus* may provide innovative options for consumption and medicinal applications in the fields of foods, dietary supplements, and biopharmaceuticals.

## 10. Challenges in Clinical Application

While the potential probiotic role of *O. splanchnicus* is promising, several challenges must be addressed before its clinical application can be realized. A panel of microbiome experts from countries in the Asia-Pacific region has reached a consensus [88], strongly recommending prioritizing the development of strains derived from human and food sources and ensuring that indications are based on clinical needs and demonstrated efficacy in research. Additionally, preclinical evaluations should encompass thorough screening, genotyping, and phenotypic analysis, as well as comprehensive in vitro and animal studies, to assess functional mechanisms and microbial safety [89]. Specifically, the safety of *O. splanchnicus* as a probiotic or therapeutic agent must be thoroughly evaluated, including assessing its potential to cause infections, particularly in immunocompromised individuals, and its interactions with other medications or existing gut microbiota, with long-term safety studies being necessary to ensure no adverse effects arise. Moreover, ensuring the viability and stability of *O. splanchnicus* during storage, transportation, and administration is crucial, as the bacterium must remain alive and active to exert its beneficial effects, which requires the development of appropriate formulation and delivery systems, such as encapsulation technologies, to protect it from harsh environmental conditions [90,91]. Additionally, the regulatory pathway for approving *O. splanchnicus* as a probiotic or therapeutic agent can be complex and time-consuming, involving meeting the requirements of regulatory agencies like the Food and Drug Administration in the United States or the European Medicines Agency in Europe, which include demonstrating safety, efficacy, and quality. Furthermore, establishing standardized protocols for the cultivation, production, and quality control of *O. splanchnicus* is essential to ensure consistency and reproducibility, encompassing defining optimal growth conditions, purification methods, and quality control measures to monitor the bacterium’s viability, purity, and potency. Lastly, the acceptance and compliance of patients with *O. splanchnicus*-based treatments or supplements are important considerations, as factors such as taste, convenience, and cost can influence patient adherence to treatment regimens, necessitating the development of palatable and user-friendly formulations to improve patient acceptance and compliance [92]. In the future, microecological preparations focusing on *O. splanchnicus* may provide innovative options for consumption and medicinal applications in the fields of foods, dietary supplements, and biopharmaceuticals; however, addressing these challenges will be critical to realizing its full potential in clinical practice.

## 11. Conclusions

Although the research on *O. splanchnicus* is still in its preliminary stages, it is a crucial member of the human gut microbiota and has emerged as a microbial species with numerous beneficial properties. Notably, it exerts direct and indirect regulatory effects in pathophysiological processes associated with diseases such as inflammatory bowel disease, NAFLD, and CF. This highlights its potential as a therapeutic agent. *O. splanchnicus* demonstrates a positive role in maintaining gut homeostasis and promoting health. However, under specific pathological conditions, such as exacerbated intra-abdominal inflammation or impaired intestinal barrier function, it may act as an opportunistic pathogen, triggering infections or even sepsis.

In terms of the limitations of this article, it is noteworthy that the association of *O. splanchnicus* with various diseases is complex and multifaceted. Our study reveals a significant correlation. It must be acknowledged that diseases are not solely attributed to the presence or absence of a single microorganism. Rather, they result from the intricate interplay of multiple factors, including diet, lifestyle, genetic background, and other environmental influences. Furthermore, the sample sizes in many relevant studies may be limited. A small sample size can introduce bias and reduce the statistical power to detect true associations or differences. With a larger and more diverse sample, we could derive more reliable and robust conclusions regarding the role of *O. splanchnicus* in different diseases. Our current understanding of *O. splanchnicus* is primarily based on correlational studies, and its precise mechanisms of action remain to be fully elucidated. The identification of *O. splanchnicus* as a potential NGP candidate is a hypothesis-driven approach that heavily relies on comparing relative abundance levels between healthy and diseased subjects. Additionally, achieving large-scale production of live biotherapeutic products containing *O. splanchnicus* poses challenges due to its uncommon growth requirements. Ensuring the viability and bioactivity of the culture until consumption is another critical aspect that needs to be addressed. Future research should focus on well-designed clinical trials to evaluate the safety and efficacy of *O. splanchnicus* in human subjects. Further in-depth studies are also necessary to elucidate its mechanisms of action and interactions with the host immune system and gut microbiota. Moreover, efforts should be directed toward optimizing the formulation and delivery of *O. splanchnicus*-based probiotics to enhance their therapeutic potential. By addressing these limitations and exploring prospects, *O. splanchnicus* holds great promise as a novel therapeutic agent for modulating gut microbiota and alleviating associated health conditions.

## Data Availability

No new data were created or analyzed in this study.

## Abbreviations

NGPs	next-generation probiotics
SCFAs	short-chain fatty acids
CRC	colorectal cancer
T1D	type 1 diabetes
aGVHD	acute graft-versus-host disease
CF	cystic fibrosis
NAFLD	non-alcoholic fatty liver disease
IBS	irritable bowel syndrome
FMT	fecal microbiota transplantation
SLE	systemic lupus erythematosus
ADHD	attention-deficit hyperactivity disorder
ACS	acute coronary syndrome
HCC	hepatocellular carcinoma
KC	kidney cancer
PDAC	pancreatic ductal adenocarcinoma
AIP	autoimmune pancreatitis
